# Integration of Aromatherapy and Essential Oils in Asthma Symptom Relief: A Narrative Review

**DOI:** 10.1002/hsr2.73425

**Published:** 2026-10-27

**Authors:** Alex Odoom

**Affiliations:** ^1^ Department of Medical Microbiology University of Ghana Medical School, Korle‐Bu Accra Ghana

**Keywords:** aromatherapy, asthma, complementary therapy, essential oils, symptom

## Abstract

**Background and Aims:**

Asthma affects 262 million people globally, causing 455,000 preventable deaths, with higher mortality in low‐resource areas due to limited diagnosis and treatment. Aromatherapy, using bioactive essential oils (EOs), has emerged as a promising complementary approach for asthma symptom management.

**Methods:**

This review synthesizes the current evidence on the therapeutic potential of EOs in alleviating asthma‐related inflammation, bronchoconstriction, and immune dysregulation through anti‐inflammatory, bronchodilatory, and immunomodulatory mechanisms.

**Results:**

Key oils such as lavender, eucalyptus, and peppermint demonstrate efficacy in reducing airway hyperresponsiveness, oxidative stress, and Th2‐mediated inflammation, supported by preclinical and clinical studies. Methods of application, including inhalation, topical use, and environmental diffusion, are evaluated for their physiological and psychological benefits, particularly in stress and sleep modulation, which are critical to asthma control. Safety considerations, such as allergenicity, proper dosing, and potential drug interactions, are emphasized to ensure safe integration with conventional therapies.

**Conclusion:**

Despite the promising findings, further large‐scale clinical trials are needed to validate the efficacy, optimize protocols, and explore personalized approaches based on genetic and phenotypic variability. This review underscores aromatherapy's role as an adjunctive therapy within a holistic asthma management framework, bridging traditional knowledge with contemporary scientific validation to improve patient outcomes.

**Abbreviations**
AEO: *Asarum sieboldii Radix* essential oil
BALF: bronchoalveolar lavage fluid
BC‐oil: *Bupleurum chinense* essential oil
BEO: bergamot essential oil
BSMC: bronchial smooth muscle cells
CAM: complementary and alternative medicine
CaMKII: calcium/calmodulin‐dependent protein kinase II
CLEO: *Chamaecyparis obtusa* leaf essential oil
EOA: *Abies holophylla* leaf essential oil
EOCZ: *Croton zehntneri* essential oil
EOPI: *Pistacia integerrima* essential oil
EOs: essential oils
EPCAM: epithelial cell adhesion molecule
FeNO: fractional exhaled nitric oxide
FEV1: forced expiratory volume in 1 s
FVC: forced vital capacity
HC: *Houttuynia cordata* essential oil
hs‐CRP: high‐sensitivity C‐reactive protein
IFN‐γ: interferon‐gamma
IL: interleukin
IP3R: inositol 1,4,5‐trisphosphate receptor
JAK: janus kinase
LPS: lipopolysaccharide
MAPK: mitogen‐activated protein kinase
MEF: maximum expiratory flow
MEO: *Mentha* essential oil
MMEF: maximum mid‐expiratory flow
NF‐κB: nuclear factor kappa‐B
NLRP3: NOD‐like receptor family pyrin domain containing 3
OVA: ovalbumin
PEF: peak expiratory flow
PFT: pulmonary function test
PM: particulate matter
PM10: particulate matter with a diameter of 10 micrometers or less
POSTN: periostin
RUNX3: Runt‐related transcription factor 3
SERCA2A: sarco/endoplasmic reticulum Ca^2+^‐ATPase 2A
SOD: superoxide dismutase
STAT: signal transducer and activator of transcription
TGF‐β: transforming growth factor‐beta
Th: T helper cells
TNF‐α: tumor necrosis factor‐alpha
TPC2: two‐pore channel 2
TRAF6: TNF receptor associated factor 6

## 1 Introduction

Aromatherapy is an integrative health modality that utilizes carefully administered plant‐derived essential oils to support both mental and physiological wellness [1]. This therapeutic intervention seeks to optimize holistic health outcomes by engaging multiple dimensions of wellness through the strategic use of botanical aromatic compounds. Essential oils (EOs) are the core components of aromatherapy, serving as the active agents responsible for its therapeutic effects. These oils are volatile compounds extracted from plants and are known for their distinct aromas and therapeutic properties [2]. The therapeutic properties of EOs stem from their intricate blend of volatile compounds, including terpenes, phenols, and esters. These compounds provide plants with adaptive advantages in their environment, such as attracting pollinators, repelling pests, and protecting against microbial infections [3]. When extracted and applied in aromatherapy, these same compounds can serve as important active principles in products intended for human treatment, offering a natural and holistic approach to healthcare [4].

Among the chronic diseases that may benefit from integrative strategies is asthma. While conventional pharmacotherapy, such as inhaled corticosteroids and bronchodilators, has significantly improved symptom control, many patients continue to experience suboptimal management due to medication side effects, limited access to care, or inadequate control of noninflammatory triggers like stress and poor sleep [5, 6, 7]. These challenges have prompted both patients and clinicians to explore complementary and alternative medicine (CAM). In this context, aromatherapy has gained increasing attention as a potential adjunctive therapy. Emerging evidence suggests that certain EOs possess anti‐inflammatory, bronchodilatory, mucolytic, antioxidant, and immunomodulatory properties that may directly address key pathophysiological features of asthma [8, 9]. Moreover, inhaled EOs act via the olfactory‐limbic pathway to exert central nervous system effects, offering a unique mechanism for mitigating stress‐ and anxiety‐induced bronchoconstriction [10, 11], a common yet often overlooked contributor to asthma exacerbations. This dual action positions aromatherapy as a compelling candidate for managing asthmatic conditions in which both physical airway constriction and psychological stress play significant roles.

Despite growing interest, the scientific validation of aromatherapy in asthma remains fragmented, with most data derived from preclinical models or small‐scale human trials. Therefore, this review aims to comprehensively synthesize current evidence on the efficacy, mechanisms of action, methods of application, and safety considerations of EOs in asthma management. It further explores the potential for personalized, integrative approaches that bridge traditional knowledge with modern biomedical science to enhance patient‐centered outcomes in a condition that continues to pose significant global health challenges.

### 1.1 Asthma in the Modern World

Asthma is a chronic airway disease characterized by significant exacerbation of bronchial spasm (bronchospasm) and airway inflammation. This condition leads to the narrowing of the airways, making it difficult to breathe. Asthma is characterized by recurrent episodes of wheezing, breathlessness, chest tightness, and coughing, particularly at night or early in the morning [12]. Exposure to fine particulate matter (PM) can intensify asthma symptoms, leading to increased airway inflammation and respiratory distress. PM, especially PM10 (particulate matter with a diameter of 10 micrometers or less), can penetrate deep into the lungs, causing airway epithelial remodeling, fibrosis, and pulmonary destruction in asthma [13]. This makes individuals with asthma particularly vulnerable to air pollution and environmental irritants. For instance, allergens such as pollen, dust mites, mold, and pet dander can provoke an immune response in the airways, leading to inflammation and bronchoconstriction. Environmental factors like air pollution, tobacco smoke, and cold air can also irritate the airways and trigger asthma symptoms [14]. Identifying and avoiding these triggers is a crucial aspect of asthma management, helping to reduce the frequency and severity of asthma attacks. Asthma represents a major global health challenge with significant implications for healthcare infrastructure and resource allocation. This chronic respiratory condition affects approximately 262 million individuals worldwide and was associated with 455,000 preventable deaths in 2019, with disproportionate mortality occurring in low‐resource settings where diagnostic and treatment gaps persist [12]. The clinical and economic burden of asthma is particularly evident in pediatric populations, where recurrent wheezing episodes demand substantial medical intervention and generate considerable healthcare expenditures. For instance, recent economic analyses reveal substantial healthcare expenditures associated with childhood asthma in the US healthcare system. Inflation‐adjusted data (2018 values) show that total direct costs reached $6.31 billion annually, with per‐patient expenditures varying between $3076 and $13,612 depending on disease severity [15]. These figures demonstrate significantly greater financial burdens for families with asthmatic children compared to those without respiratory conditions. The comprehensive costs of asthma management encompass direct medical expenses (including hospitalizations and pharmacotherapy) and indirect costs related to lost productivity [16]. Implementation of evidence‐based control strategies remains crucial for mitigating the societal and individual consequences of this prevalent condition.

## 2 Bioactive Phytochemicals and Their Therapeutic Effects

The therapeutic potential of essential oils stems from their diverse phytochemical composition, as illustrated in Figure 1. Table 1 summarizes the essential oils used in asthma management, including their key bioactive phytochemicals and multifaceted therapeutic mechanisms.

**Figure 1:**
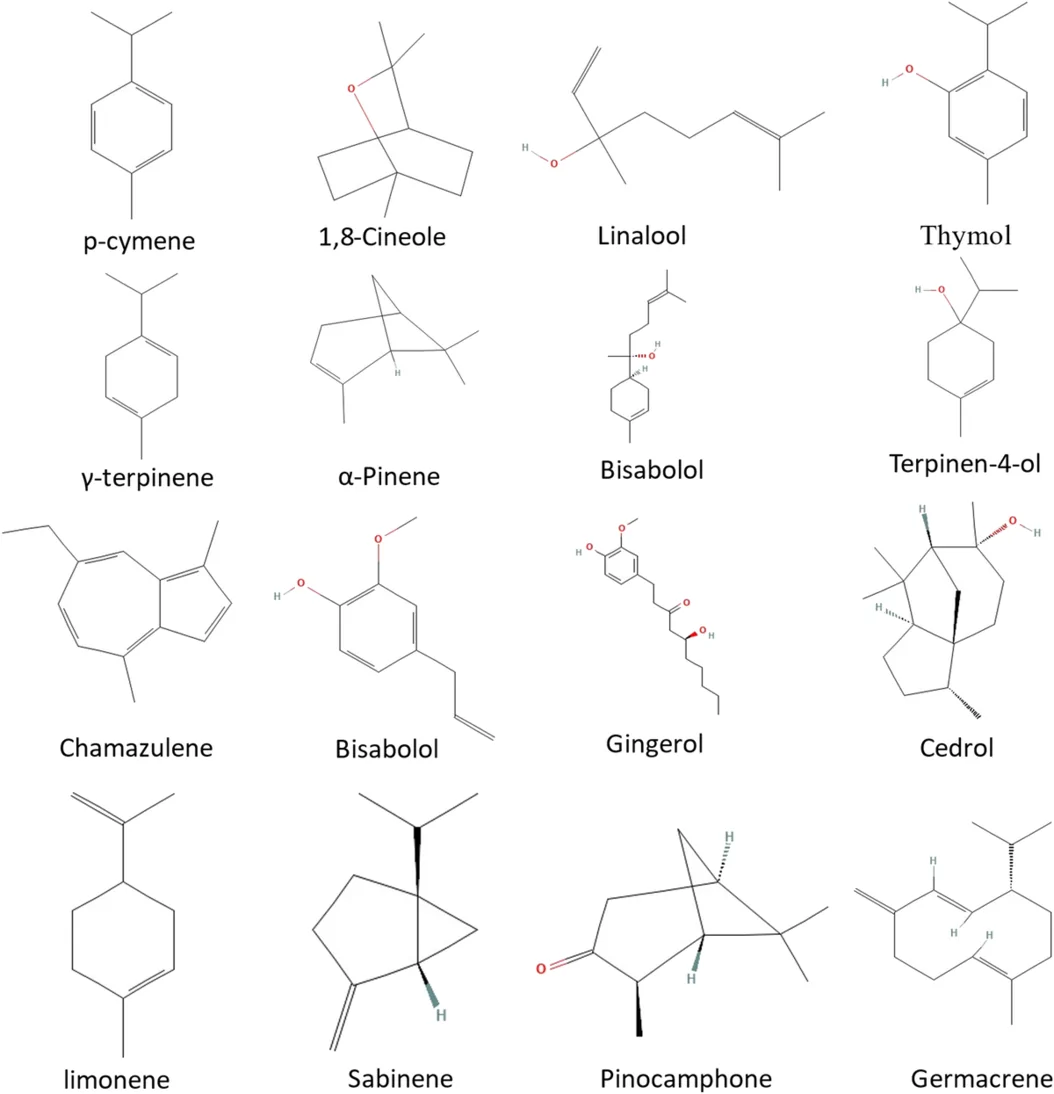
Chemical composition of essential oils.

**Table 1:** Clinically and traditionally used essential oils for asthma symptom management.

Essential oil	Key compounds	Therapeutic effects	References
Eucalyptus	1,8‐Cineole (Eucalyptol)	Bronchodilation, anti‐inflammatory, decongestant	Shiekh et al. [17], Hoch et al. [18], Soyingbe et al. [19]
Lavender	Linalool, Linalyl acetate	Anti‐anxiety, anti‐inflammatory, sedative	Donelli et al. [20], Ueno‐Iio et al. [21], Yoo and Park [22]
Peppermint	Menthol, Menthone	Bronchodilation, cooling effect, decongestant	Kim et al. [23]
Thyme	Thymol, Carvacrol	Expectorant, antimicrobial, antispasmodic	Alavinezhad et al. [24, 25]
Frankincense	α‐Pinene, Limonene	Anti‐inflammatory, immune modulation	Vora et al. [4], Salehi et al. [26]
Bergamot	Limonene, Linalool	Stress relief, anti‐inflammatory	Feng et al. [27]
Chamomile (Roman)	Bisabolol, Chamazulene	Anti‐allergic, calming, anti‐inflammatory	Fung et al. [28]
Tea tree	Terpinen‐4‐ol	Antimicrobial, anti‐inflammatory	Aswandi and Kholibrina [29], Lin et al. [30]
Rosemary	1,8‐Cineole, Camphor	Expectorant, bronchodilation	Hoch et al. [18]
Clove	Eugenol	Analgesic, antimicrobial	Fung et al. [28], Aswandi and Kholibrina [29]
Ginger	Gingerol, Zingiberene	Anti‐inflammatory, warming	Vora et al. [4]
Cedarwood	Cedrol	Sedative, anti‐inflammatory	Martins et al. [31]
Ravintsara	1,8‐Cineole	Antiviral, expectorant	Hoch et al. [18]
Cypress	α‐Pinene, Limonene	Antispasmodic, decongestant	Kim et al. [32]
Marjoram	Terpinene‐4‐ol, Sabinene	Antispasmodic, bronchodilator	Vora et al. [4]
Pine	α‐Pinene	Expectorant, anti‐inflammatory	Salehi et al. [26], Bhardwaj et al. [33]
Cardamom	Cineole, Limonene	Bronchodilator, anti‐inflammatory	Khan et al. [34]
Hyssop	Pinocamphone	Expectorant, antispasmodic	Reis and Jones [1], Vora et al. [4]
Neroli	Linalool, Nerolidol	Anti‐anxiety, anti‐inflammatory	Liang et al. [2]
Ylang‐Ylang	Linalool, Germacrene	Anti‐anxiety, bronchodilator	Fung et al. [28]

### 2.1 Anti‐Inflammatory Pathways

Essential oils demonstrate significant potential in managing asthma symptoms through multiple anti‐inflammatory pathways (Figure 2). These natural compounds modulate cytokine production and inhibit key inflammatory cascades involved in asthma pathogenesis, thereby reducing airway inflammation. Research indicates that essential oils from plants like *Canarium indicum* L., containing bioactive components such as linalool, *p*‐cymene, and *γ*‐terpinene, effectively lower malondialdehyde (MDA) levels, a marker of oxidative stress frequently elevated in asthmatic patients [35]. By decreasing lipid peroxidation, these oils mitigate tissue damage and inflammation associated with asthma. Furthermore, essential oils like *Chamaecyparis obtusa* leaf essential oil (CLEO) specifically target Th2‐mediated immune responses, significantly reducing the levels of pro‐inflammatory cytokines (IL‐4, IL‐5, and IL‐13) and IgE while decreasing eosinophil infiltration in the airways [36]. This multitargeted approach not only alleviates bronchial hyperresponsiveness but also addresses the underlying immunological dysregulation characteristic of allergic asthma, highlighting their potential as complementary anti‐inflammatory agents.

**Figure 2:**
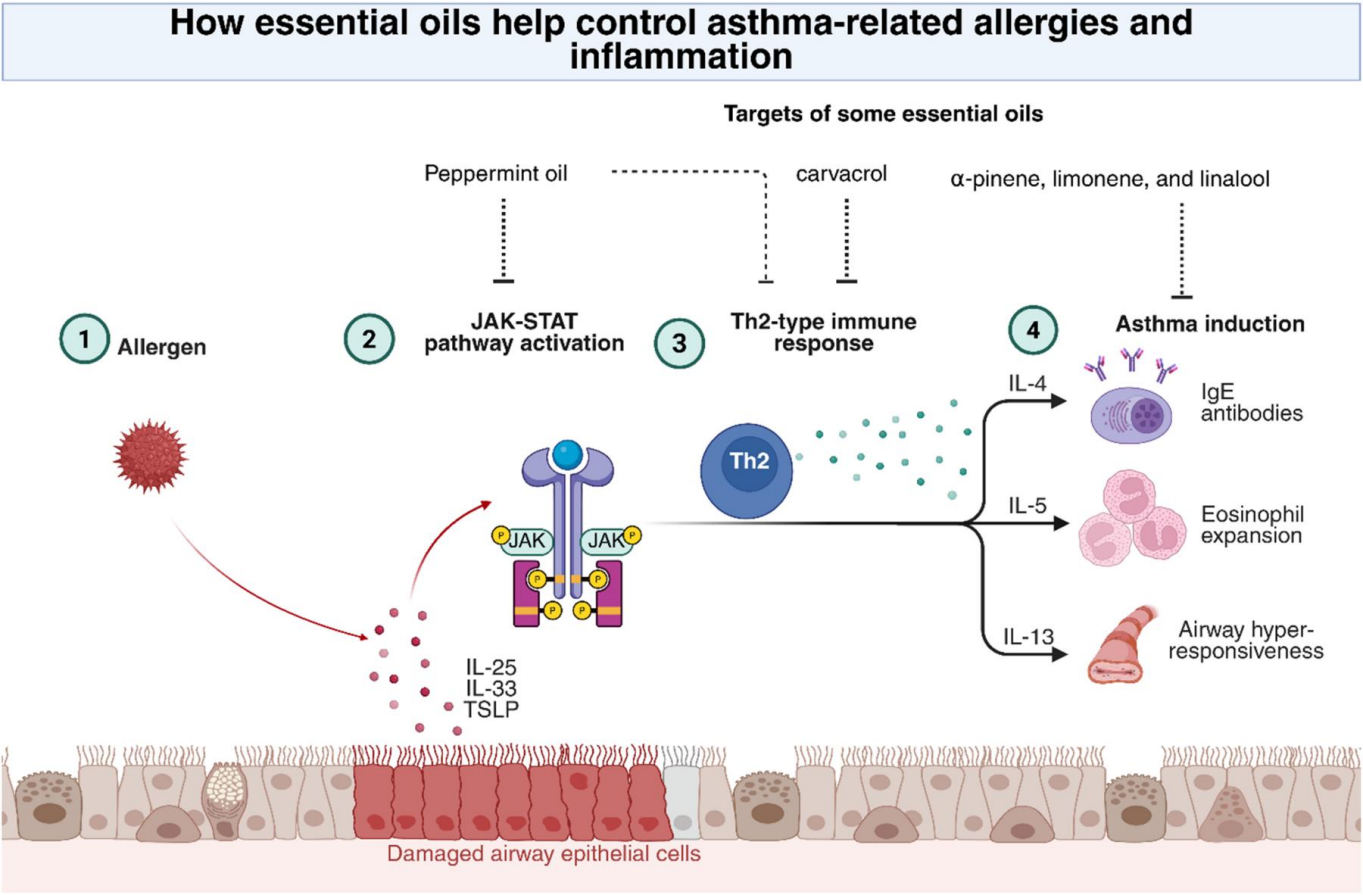
Mechanisms by which essential oils modulate allergic responses and inflammation in asthma.

### 2.2 Bronchodilation and Airway Clearance Mechanisms of Essential Oils

Essential oils demonstrate significant therapeutic potential in improving respiratory function through multiple mechanisms (Figure 2). Inhalation of eucalyptus‐based essential oils has been shown to effectively clear airway congestion and alleviate symptoms associated with respiratory conditions, including asthma, coughs, and sinusitis [17]. These benefits are frequently harnessed through inhalation therapies and topical applications like chest rubs, which help reduce bronchial constriction and facilitate easier breathing. The lipophilic properties of essential oils enable them to interact with cellular membranes, potentially disrupting viral particles that can trigger asthma exacerbations. This membrane interaction, combined with the synergistic action of multiple bioactive compounds, contributes to both antiviral effects and respiratory benefits, including bronchodilation and reduced mucus viscosity [37]. Among the key active components, 1,8‐cineole, found in various medicinal plants, including *Eucalyptus* species, *Melaleuca cajuput*, and *Cinnamomum zeylanicum*, plays a particularly important role in relaxing the airway smooth muscle tissue [18]. This monoterpene, along with other phytochemicals such as α‐pinene, limonene, and linalool, reduces bronchospasm and improves overall pulmonary function [38, 39], offering a multifaceted approach to managing asthma symptoms and maintaining airway patency.

### 2.3 Immunomodulatory Effects of Essential Oils

Essential oils exhibit significant immunomodulatory properties that can help regulate the allergic responses and inflammation associated with asthma (Figure 2). Research indicates that these botanical extracts effectively lower pro‐inflammatory markers while enhancing anti‐inflammatory mediators, offering a promising complementary approach for managing immune‐related respiratory conditions [9]. Specific essential oils like *Mentha piperita* (peppermint) modulate key inflammatory pathways involved in pollution‐induced asthma. Studies reveal that peppermint oil targets the IL‐6/JAK2/STAT3 signaling cascade, significantly reducing the levels of pro‐inflammatory cytokines, particularly those produced by Th2 cells. This mechanism involves decreased phosphorylation of JAK2 and STAT3 proteins, leading to attenuated inflammatory responses in the PM10‐exposed airways [23]. Such targeted immunomodulation helps mitigate asthma symptoms aggravated by environmental pollutants, demonstrating the potential of essential oils to address both systemic and environmental triggers of respiratory inflammation.

### 2.4 Therapeutic Synergy in Essential Oil Combinations

The multicomponent nature of essential oils creates synergistic interactions that enhance their medicinal properties beyond what single compounds can achieve. The complex interplay between these bioactive molecules is summarized in Table 2.

**Table 2:** Mechanisms of therapeutic synergy in essential oil combinations.

Synergy category	Mechanism of action	Clinical/therapeutic benefit	References
Whole‐plant interaction	Interaction of numerous bioactive molecules working in concert to target different phases of pathogenic processes.	Provides more complete symptom relief compared to isolated components; facilitates bronchodilation and mucolytic action.	Bunse et al. [40]
Multisystem modulation	Strategic blending of diverse phytochemical profiles to simultaneously modulate several physiological systems.	Combines antioxidant protection with anti‐inflammatory and antimicrobial activities to manage inflammation and bronchial constriction.	Edo et al. [41]
Aromatherapeutic Pairing	Integration of anxiolytic (anti‐anxiety) properties with direct respiratory support.	Significant improvement in patients experiencing nocturnal breathing difficulties and anxiety‐related respiratory distress.	Hedigan et al. [42]

## 3 Scientific Studies and Clinical Trials: Evaluating the Efficacy of Aromatherapy for Asthma

Aromatherapy has emerged as a promising complementary approach for asthma symptom management, providing a natural adjunct to conventional medical therapies without replacing them. This integrative treatment modality leverages the therapeutic properties of specific essential oils to address characteristic asthma symptoms, including wheezing, dyspnea, and persistent coughing.

### 3.1 Human Clinical Trials

Recent clinical trials have demonstrated the therapeutic potential of plant‐derived compounds for treating asthma (Table 3). Carvacrol, a monoterpene, was evaluated in a randomized controlled trial involving patients with asthma [24]. After 2 months of treatment, the carvacrol group showed significant improvements in pulmonary function (FVC, MMEF, MEF), reduced respiratory symptoms (daytime wheezing, exercise‐induced symptoms), and decreased inflammatory markers (hs‐CRP, eosinophil counts). These findings suggest that carvacrol's anti‐inflammatory properties may underlie its clinical benefits. A subsequent study reinforced these results, showing that carvacrol additionally improved oxidative stress markers (reduced MDA, increased SOD) and rebalanced Th1/Th2 cytokines (elevated IFN‐γ, decreased IL‐4), further supporting its multimodal mechanism of action [43].

**Table 3:** Human clinical trials on essential oils/compounds for asthma management.

Compound/extract	Target	Mechanism of action	Study design	Key findings	Development stage	References
Carvacrol	Asthma	Anti‐inflammatory properties	Randomized, double‐blind, placebo‐controlled, Phase II	Improved PFT values (FVC, MMEF, MEF) Reduced respiratory symptoms (wheezing) Decreased inflammatory markers (WBC, eosinophils, monocytes, hs‐CRP)	Phase II	Alavinezhad et al. [24]
Carvacrol	Asthma	Reduced oxidative stress Modulated Th1/Th2 cytokine balance	Randomized, double‐blind, placebo‐controlled	Improved PFT values (FVC, PEF, MMEF) Reduced respiratory symptoms (wheezing, cough) Decreased oxidants, increased antioxidants Increased IFN‐γ, decreased IL‐4	Phase II	Ghorani et al. [43]
*Zataria multiflora*	Asthma	Modulated oxidative stress Modulated immune system (Th1/Th2 balance)	Randomized, double‐blind, placebo‐controlled, Phase II	Improved clinical symptoms and PFT values Reduced oxidative stress markers Increased anti‐inflammatory cytokine IFN‐γ, decreased pro‐inflammatory cytokine IL‐4	Phase II	Alavinezhad et al. [25]
Purifying air spray with 41 essential oils	Allergic asthma	No significant effects on airway inflammation, lung function, or asthma control	Prospective open‐label study	No changes in FeNO, lung function, airway hyperresponsiveness, or asthma control No significant changes in most respiratory symptoms, except an increase in odor perception and a decrease in eye irritation	Early stage	Levy et al. [44]

*Zataria multiflora* (containing thymol/carvacrol) was evaluated in a similar trial design [25]. The treatment group exhibited enhanced lung function (FEV1, MEF parameters), reduced nocturnal symptoms, and improved oxidative/immune profiles, including elevated antioxidant enzymes (catalase, SOD) and a shifted IFN‐γ/IL‐4 ratio via PBMC gene modulation. These studies collectively highlight how phytochemicals can simultaneously target inflammation, oxidative stress, and immune dysregulation in asthma. In contrast, the safety of an essential oil‐based air spray was assessed in patients with allergic asthma [44]. No significant changes occurred in FeNO, lung function, or symptom scores after the 4‐week exposure, suggesting tolerability in controlled asthma. However, increased odor perception was noted, emphasizing the need for individualized approaches in essential oil therapies.

### 3.2 Animal Studies and Preclinical Research

#### 3.2.1 Modulation of the Inflammatory Pathways

Multiple studies have demonstrated that essential oils can effectively mitigate asthma‐related inflammation through various molecular mechanisms (Table 4). In a murine asthma model, lavender oil (Lvn) inhalation demonstrated significant anti‐inflammatory effects, including reduced airway hyperresponsiveness and decreased inflammatory cell infiltration in lung tissues and BAL fluid. The treatment notably suppressed Th2 cytokine production (IL‐5, IL‐13) and Muc5b expression, suggesting its potential for managing allergic airway inflammation [21]. Moreover, the effects of *Abies holophylla* leaf essential oil (EOA) on airway inflammation were examined using an asthma model [45]. Network pharmacology analysis linked EOA to the IL‐17 signaling pathway, a key player in asthma pathogenesis. In vivo experiments demonstrated that EOA inhalation reduced respiratory epithelial hyperplasia, collagen deposition, and goblet cell activation while decreasing inflammatory cells (eosinophils, lymphocytes, macrophages) in the bronchoalveolar lavage fluid (BALF). Additionally, EOA suppressed Th2 cytokines (IL‐4, IL‐5, and IL‐13) and IgE production by inhibiting IL‐17‐related pathways, including TRAF6‐NF‐κB and MAPK cascades, and modulating the Th17/Treg balance.

**Table 4:** Preclinical studies on essential oils and their antiasthmatic effects.

Essential oil	Target	Mechanism of action	Model	References
Lavender (Lvn)	Allergic airway inflammation	Suppressed airway hyperresponsiveness, inflammatory cell accumulation, and mucous cell hyperplasia; Decreased Th2 cytokines (IL‐5, IL‐13)	Mouse model of allergic asthma	Ueno‐Iio et al. [21]
Safranal (from *Crocus sativus*)	Airway inflammation, hyperreactivity, and remodeling	Modulated NLRP3/caspase‐1 pathway to restore RUNX3‐mediated immunoregulation	In vitro ASMC model and mouse model of allergic asthma	Zhang et al. [46]
*Abies holophylla* leaf (EOA)	Airway inflammation	Inhibited IL‐17‐related signaling pathways (TRAF6–NF‐κB, MAPK); Maintained Th17/Treg cell balance	Mouse model of allergic asthma	Park et al. [45]
Bergamot (BEO)	Airway inflammation and remodeling	Modulated MAPK and JAK–STAT signaling pathways	Mouse model of allergic asthma, in vitro macrophage model	Feng et al. [27]
*Asarum sieboldii Radix* (AEO)	Airway inflammation, remodeling, and fibrosis	Regulated POSTN/TGF‐β‐related pathways	Mouse model of OVA‐ and PM10‐induced asthma	Han et al. [47]
*Mentha* (MEO)	Particulate matter (PM10)‐exposed asthma	Suppressed IL‐6/JAK2/STAT3 signaling pathway	In vitro PM10‐exposed lung epithelial cells in a mouse model of PM10‐induced asthma	Kim et al. [23]
*Pistacia integerrima* (EOPI)	Airway inflammation and hyperresponsiveness	Inhibited 5‐lipoxygenase, mast cell stabilization, L‐type calcium channel modulation, antioxidant, and anti‐angiogenic activities	In vitro models, guinea pig model of OVA‐induced bronchoconstriction, rat model of LPS‐induced lung inflammation	Shirole et al. [48]
Lauraceae family (*Cinnamomum*, *Lindera*, *Machilus*, *Neolitsea*)	Airway inflammation and mucus hypersecretion	Suppressed pro‐inflammatory cytokines (IL‐1β, IL‐6) and mucin genes (MUC5AC, MUC5B)	In vitro model of LPS‐stimulated bronchial epithelial cells	Yang et al. [49]
*Houttuynia cordata* (HC)	Airway remodeling	Modulated intracellular calcium (Ca^2+^) signaling, including IP3R, TPC2, and SERCA2A	In vitro model of IL‐4/IL‐13‐treated bronchial smooth muscle cells, a mouse model of house dust mite‐induced asthma	Huang et al. [50]
*Croton tentneri* (EOCZ)	Lung injury	Reduced oxidative stress markers (catalase, SOD)	Mouse model of OVA‐induced asthma	Serra et al. [51]
*Eucalyptus grandis*	Asthma and cough	Anti‐inflammatory activity and vasorelaxant effects on smooth muscle	Experimental animal models	Soyingbe et al. [19]
*Bupleurum chinense* DC. (BC‐oil)	Asthma	Modulated ectopic olfactory receptor OR2W3 and the downstream CaMKII signaling pathway	In vivo rat model of asthma and in vitro cell line studies	Li et al. [52]
*Zanthoxylum armatum*	Asthma	Bronchodilatory effects Reduced eosinophils, neutrophils, and IgE levels Improved lung histopathology	Guinea pig model of histamine‐induced bronchoconstriction and mouse model of OVA‐induced asthma	Sharma et al. [53]

*Mentha* essential oil (MEO) was investigated in a particulate matter (PM10)‐induced asthma model, combining network pharmacology with in vitro and in vivo analyses [23]. Computational predictions identified cytokine–cytokine receptor interactions and JAK–STAT signaling as the key pathways targeted by MEO. In asthmatic mice, MEO inhalation attenuated PM10‐induced airway remodeling, collagen accumulation, and goblet cell hyperplasia. Cellular studies revealed that MEO downregulated pro‐inflammatory (IL‐6, TNF‐α, IL‐1β) and Th2‐associated cytokines (IL‐4, IL‐13) in PM10‐exposed A549 lung cells. Mechanistically, MEO inhibited JAK2/STAT3 activation and suppressed NF‐κB signaling by reducing IκB‐α phosphorylation and nuclear translocation of NF‐κB. These findings suggest that MEO alleviates PM10‐aggravated asthma by modulating the IL‐6/JAK2/STAT3 axis, offering a rationale for its use in the treatment of pollution‐related respiratory inflammation.

#### 3.2.2 Immunomodulatory and Antioxidant Effects

Essential oils demonstrate significant immunomodulatory potential in asthma management. Recently, safranal from saffron has been identified as an effective compound against asthma pathogenesis [46]. Research revealed that safranal restores RUNX3 expression in TNF‐α–stimulated airway smooth muscle cells, thereby mitigating inflammation and oxidative stress. In vivo studies showed safranal's ability to alleviate asthma symptoms through NLRP3/Caspase‐1 pathway modulation, with diminished effects in RUNX3‐deficient mice, highlighting its mechanism of action [46].

Similarly, bergamot essential oil (BEO), with d‐limonene and *γ*‐terpinene as major components, was investigated [27]. Network analysis suggested that BEO's anti‐asthma effects involve the MAPK and JAK–STAT pathways. In an OVA‐induced asthma model, BEO inhalation alleviated lung inflammation and airway remodeling while reducing Th2 cytokine and IgE levels. In vitro studies confirmed BEO's suppression of inflammatory mediators (IL‐6, IL‐1β, TNF‐α) in macrophages via MAPK and JAK–STAT inhibition, supporting its potential as a natural asthma treatment. Moreover, *Asarum sieboldii Radix* essential oil (AEO) was studied, and its primary compound, methyl eugenol, was found to share 48% of the asthma‐related gene targets [47]. In OVA/PM10‐induced asthma, AEO reduced airway remodeling, inflammatory cell infiltration, and IgE/IgG2a levels while downregulating pro‐inflammatory and Th2 cytokines. AEO also inhibited fibrotic mediators (POSTN, TGF‐β) and epithelial–mesenchymal transition markers, suggesting that its antifibrotic effects occur via POSTN/TGF‐β pathway modulation. These findings highlight AEO's potential in mitigating asthma‐related inflammation and fibrosis.

#### 3.2.3 Mucoregulatory

Essential oils demonstrate significant potential in addressing two critical aspects of asthma pathophysiology: mucus hypersecretion and secondary infections. A comparative analysis of essential oils from seven Lauraceae species revealed distinct phytochemical profiles. *Cinnamomum camphora* oil was rich in camphor (89.09%), while *Lindera obtusiloba* oil contained d‐limonene (10.27%) and β‐eudesmol (10.03%) [49]. Noncytotoxic concentrations of these oils suppressed IL‐1β and IL‐6 in LPS‐stimulated NCI‐H292 bronchial cells, with *L. obtusiloba* oil showing the highest efficacy. Notably, *L. obtusiloba* oil revealed potent mucoregulatory effects through the suppression of MUC5AC and MUC5B gene expression in bronchial epithelial cells. Other oils, such as those from *C. cassia* (linalool) and *C. japonicum* (1,8‐cineole), exhibited moderate anti‐inflammatory activity. This study underscores the potential of Lauraceae essential oils, particularly *L. obtusiloba*, as natural therapeutics for asthma‐associated mucus hypersecretion and inflammation.

#### 3.2.4 Bronchodilation and Airway Remodeling

Several essential oils have shown promise in improving respiratory function and preventing airway remodeling. *Eucalyptus grandis* essential oil and its primary component, 1,8‐cineole, were investigated, revealing significant anti‐asthmatic and antitussive properties in rodent models [19]. Both substances exhibited dose‐dependent effects in delaying asthma‐associated symptoms (wheezing, coughing) and demonstrated anti‐inflammatory activity in the cotton pellet granuloma assay. Notably, they induced vasorelaxation in rat aortic smooth muscle, suggesting potential bronchodilatory effects through direct smooth muscle relaxation, supporting their traditional use in respiratory therapeutics. Similarly, *Pistacia integerrima* essential oil (EOPI) was examined, demonstrating its multitarget antiasthmatic effects [48]. In vitro assays revealed EOPI's 5‐lipoxygenase inhibitory activity, antioxidant capacity (DPPH scavenging), and mast cell‐stabilizing properties. Ex vivo experiments on the guinea pig ileum showed EOPI‐mediated relaxation via L‐type calcium channel blockade, as evidenced by its inhibition of acetylcholine‐, histamine‐, and Bay K 8644‐induced contractions. EOPI also exhibited anti‐angiogenic effects in a chick chorioallantoic membrane model, suggesting its potential to mitigate airway remodeling. In vivo, EOPI reduced LPS‐induced pulmonary inflammation in rats by lowering inflammatory cell influx, myeloperoxidase activity, and oxidative stress markers in the bronchoalveolar fluid. In addition, it prevented ovalbumin (OVA)‐induced bronchoconstriction in sensitized guinea pigs, highlighting its dual anti‐inflammatory and bronchodilatory effects. These results align with the traditional applications of *P. integerrima* in respiratory disorders, underpinned by its 5‐lipoxygenase inhibition, calcium channel modulation, and antioxidant actions.

Moreover, *Croton zehntneri* essential oil (EOCZ) was evaluated in OVA‐induced asthma, demonstrating beneficial effects on lung mechanics and oxidative stress [51]. OVA‐challenged mice exhibited impaired respiratory function (elevated airway resistance, tissue elastance) and structural changes (alveolar collapse, inflammation), which were reversed by high‐dose EOCZ (300 mg/kg). EOCZ also normalized oxidative stress markers (catalase and superoxide dismutase), suggesting its dual anti‐inflammatory and antioxidant mechanisms. These findings support EOCZ as a complementary option for asthma management. Furthermore, *Houttuynia cordata* (HC) essential oil was investigated for its effects on calcium‐mediated airway remodeling [50]. In interleukin‐4/13–stimulated human bronchial smooth muscle cells (hBSMCs), increased expression of calcium channels (IP3R1, TPC2, and SERCA2A) and enhanced IP3‐mediated calcium release were observed, suggesting their involvement in asthma pathogenesis. HC essential oil pretreatment dose‐dependently reduced ATP‐induced calcium release in hBSMCs. In a house dust mite‐induced murine asthma model, nebulized HC essential oil attenuated airway inflammation (reduced neutrophils/eosinophils) and remodeling markers (airway thickness, smooth muscle hyperplasia), indicating its potential as an adjunct therapy for asthma. *Zanthoxylum armatum* essential oil, rich in linalool, has antiasthmatic properties [53]. In guinea pig and murine models, it prolonged histamine‐induced bronchoconstriction latency and reduced asthma biomarkers (IgE, eosinophils).

#### 3.2.5 Novel Mechanisms

Emerging research has revealed innovative mechanisms of action. For instance, *Bupleurum chinense* essential oil (BC‐oil) was explored in an OVA‐induced asthma model, and oral administration was shown to improve lung function and reduce inflammatory markers (IL‐2, eosinophils) [52]. Transcriptomic analysis implicated the olfactory receptor OR2W3 and CaMKII signaling in the BC‐oil mechanism. GC–MS identified linoleic acid as a key component capable of activating OR2W3, highlighting a novel pathway for essential oil‐mediated asthma relief. These findings bridge traditional use with modern pharmacology, suggesting that BC‐oil modulates ectopic olfactory receptors to exert anti‐inflammatory and antiproliferative effects in airway cells.

## 4 Methods of Application

### 4.1 Inhalation Methods

Aromatherapy inhalation harnesses the olfactory‐limbic connection to influence physiological and psychological states, with volatile compounds modulating breathing patterns via nasal chemoreceptors [4, 11, 54]. Clinical evidence supports these dual benefits; patients, including those with COVID‐19, reported improved respiratory function alongside reduced dyspnea and anxiety [55]. A randomized trial further validated this, showing that damask rose oil significantly lowered anxiety and improved sleep compared to placebo, supporting its role in integrative care [56]. Additionally, nebulization offers targeted delivery to deep lung tissue. Research on *M. piperita* demonstrated that inhalable aerosols reduced asthma pathologies, including collagen deposition and goblet cell metaplasia, by modulating the JAK/STAT inflammatory pathway [23].

### 4.2 Topical Applications

Essential oils penetrate the skin to exert systemic healing effects, a process often complemented by the stimulation of olfactory receptors through inhalation. To ensure safety and improve absorption, these oils are typically diluted with carrier oils, which minimize the risk of skin irritation [57, 58]. Using diluents like fractionated coconut oil helps distribute the oils evenly and reduces the likelihood of adverse reactions [30]. Beyond systemic absorption, topical application provides targeted relief for muscle discomfort and promotes wound healing due to its antimicrobial and anti‐inflammatory properties [17, 59]. Furthermore, chest rubs can clear airways and alleviate congestion in respiratory conditions like asthma, making this dual topical and aromatic approach a valuable strategy for integrative wellness.

### 4.3 Environmental Diffusion and Air Purification

While essential oil air sprays are popular, their safety for asthmatics requires caution due to limited research on potential bronchial irritation [44]. Conversely, diffusers provide effective, controlled exposure that facilitates stress modulation via the olfactory system [60]. This method offers dual benefits; botanicals like lavender and bergamot exhibit anxiolytic properties that mitigate stress, potentially improving respiratory outcomes given the link between emotional distress and asthma exacerbations [28].

## 5 Safety Considerations and Potential Risks

### 5.1 Allergic Reactions and Sensitization Risks

While essential oils are generally regarded as safe at appropriate dilutions, clinical evidence confirms their notable allergenic potential, necessitating careful usage protocols [4]. Essential oils carry a significant risk of allergic reactions, particularly in individuals with sensitive skin or pre‐existing allergies. The complex chemical composition of these plant‐derived extracts includes numerous bioactive compounds that may trigger contact dermatitis, respiratory irritation, or other hypersensitivity responses [61], underscoring the importance of preliminary patch testing before broader application. This precautionary approach helps identify potential allergens while minimizing the risk of severe dermatological or respiratory reactions, particularly in vulnerable populations.

### 5.2 Importance of Proper Dosage and Concentration in Essential Oil Use

Regulating essential oil concentration is vital to minimize adverse effects, particularly for individuals with asthma, as improper use can intensify respiratory symptoms. High concentrations or undiluted application can trigger reactions ranging from skin irritation to respiratory discomfort [62]. Therefore, application requires well‐ventilated spaces and adherence to strict safety guidelines. For topical use, dilution with carrier oils is essential to reduce skin sensitivity; these diluents lower potency while facilitating gradual absorption [30]. Proper dilution ratios must always be tailored to individual needs to ensure safe therapeutic outcomes.

### 5.3 Potential Drug Interactions With Essential Oils in Asthma Treatment

Essential oils may interact with asthma medications, potentially altering their efficacy or side effects. Consequently, patients should consult healthcare providers to ensure safe integration and avoid unintended consequences [58]. While aromatherapy offers complementary well‐being benefits, the current understanding of drug interactions is limited. Further research is therefore required to clarify pharmacological effects and establish evidence‐based guidelines for using essential oils concurrently with conventional treatments.

## 6 The Psychological Impact of Aromatherapy on Asthma Management

Aromatherapy is increasingly recognized not only for its physiological effects but also for its psychological benefits in chronic respiratory conditions like asthma. As summarized in Table 5, essential oils have demonstrated clinically relevant effects in reducing anxiety, improving sleep quality, and enhancing patients' coping strategies.

**Table 5:** The psychological impact of aromatherapy on asthma management.

Impact area	Mechanism of action	Key essential oils	Clinical outcomes	References
Anxiety and stress reduction	Olfactory stimulation of the limbic system regulates emotions and minimizes emotional triggers.	Lavender, bergamot, sweet orange (relaxation); peppermint (pain relief)	Reduced stress levels; improved symptom control and quality of life.	Fung et al. [28]
Improved sleep quality	Mitigates the inflammatory and immune‐compromising effects of poor sleep.	Lavender, bergamot	Alleviation of mild depression and night‐time anxiety; improved respiratory function during sleep.	Karadag et al. [63], Toprak et al. [64]
Enhanced coping mechanisms	Fosters self‐efficacy and a sense of control over the health condition.	General aromatherapy usage	Increased optimism; better adherence to conventional treatment; improved overall wellness.	Vora et al. [4]

## 7 Future Directions and Research Needs

### 7.1 Integrative and Personalized Approaches

Aromatherapy functions best as a supportive complement to standard asthma treatments rather than a replacement. Effective management integrates medication, lifestyle modifications, and complementary therapies to address both physiological and psychological aspects of the condition [65]. This holistic strategy relies on patient education and close collaboration with healthcare providers to develop personalized plans and monitor progress [66]. Future research should prioritize customizing regimens based on genetic biomarkers to improve therapeutic precision. Clinical applications must leverage aromatherapy's stress‐modulating capabilities [67], while ensuring treatments are individualized according to genetic and environmental factors to achieve optimal results [68].

### 7.2 Large‐Scale Clinical Trials

To establish aromatherapy as a credible asthma therapy, rigorous large‐scale trials are required to assess its efficacy, safety, and long‐term impacts. Research must identify optimal application methods and essential oil combinations tailored to different patient phenotypes [69, 70]. While preliminary studies suggest benefits for symptom relief, sleep, and emotional well‐being, comprehensive evidence is necessary to validate these findings and establish standardized treatment protocols.

### 7.3 Investigating Specific Mechanisms of Action

Comprehensive research is required to delineate the specific biological pathways and cellular targets of essential oils to optimize therapeutic applications [71]. Emerging evidence highlights immunomodulatory properties relevant to respiratory conditions [72], with *Curcuma* species demonstrating significant anti‐inflammatory potential [73]. Furthermore, advanced computational methods are currently being employed to analyze bioactive interactions with inflammatory pathways, potentially establishing a foundation for novel complementary treatments [74].

## 8 Concluding Remarks

Aromatherapy and essential oils present a valuable complementary approach for asthma management, demonstrating potential benefits in symptom relief, inflammation reduction, and overall wellness enhancement. While existing studies offer encouraging evidence, additional rigorous research is necessary to establish standardized protocols regarding the effectiveness, safety profiles, and ideal administration methods. When incorporated as part of a comprehensive treatment strategy under medical supervision, aromatherapy may contribute to improved disease control and quality of life for patients with asthma.

## Author Contributions

**Alex Odoom:** conceptualization, methodology, investigation, funding acquisition, writing – original draft, validation, visualization, writing – review and editing, software, formal analysis, project administration, data curation, supervision, resources. The author has read and approved the final version of the manuscript.

## Funding

The author has nothing to report.

## Disclosure

Alex Odoom affirms that this manuscript is an honest, accurate, and transparent account of the study being reported; that no important aspects of the study have been omitted; and that any discrepancies from the study as planned (and, if relevant, registered) have been explained.

## Ethics Statement

The author has nothing to report.

## Consent

The author has given consent for the publication of this manuscript.

## Conflicts of Interest

The author declares no conflicts of interest.

## Data Availability

Data sharing is not applicable to this article as no data sets were generated or analyzed during the current study. Alex Odoom had full access to all of the data in this study and takes complete responsibility for the integrity of the data and the accuracy of the data analysis.
